# COVID‐19 vaccination in patients with multiple sclerosis: what you need to know – a review

**DOI:** 10.1002/hsr2.70119

**Published:** 2024-10-06

**Authors:** Farhad Mahmoudi, Omid Mirmosayyeb, Elnaz Shaabani, Elham Moases Ghaffary, Flavia Nelson

**Affiliations:** ^1^ Department of Neurology University of Miami Miami Florida USA; ^2^ Department of Neurology, Jacobs School of Medicine and Biomedical Sciences University at Buffalo, State University of New York Buffalo New York USA; ^3^ Koch Institute for Integrative Cancer Research at MIT Massachusetts USA; ^4^ Pharmacy School University of Missouri‐Kansas City Kansas City Missouri USA

**Keywords:** COVID‐19, COVID‐19 vaccine, disease‐modifying therapies, immune response, Multiple Sclerosis, vaccine safety

## Abstract

**Background:**

The appearance of severe acute respiratory syndrome Coronavirus 2 (SARS‐CoV‐2) initiated the COVID‐19 pandemic, resulting in millions of confirmed cases and numerous fatalities. In response, rapid vaccine development efforts were launched to mitigate the pandemic's impact. Despite the high efficacy of COVID‐19 vaccines, they are also associated with several common side effects/complications, some of them specific to the multiple sclerosis population. Our goal is to review various types of COVID‐19 vaccines, assessing their efficacy, adverse events, their association with an MS relapse following vaccination, and the influence of disease modifying therapies (DMTs) on vaccines’ efficacy.

**Methods:**

The review was based on a database search that included PubMed/Medline, Embase, Scopus, and the Web of Science conducted from January 2020 to July 2024 using the following MeSH terms: MS, COVID‐19, COVID‐19 vaccination, vaccine side effects, and vaccine hesitancy.

**Results:**

Receiving any type of COVID‐19 vaccine is a safer and more reliable approach to building immunity compared to becoming infected with the virus. Complications tend to be mild to moderate, occasionally severe. DMTs could affect the humoral response to the COVID‐19 vaccine. Among all DMTs, a notable reduction in the humoral response has been observed in patients who received anti‐CD20 and sphingosine‐1‐phosphate (S1P) receptor modulator drugs after their COVID‐19 vaccination.

**Conclusion:**

Despite certain drawbacks, the benefits of the COVID‐19 vaccine significantly outweigh the associated risks, making it a recommended course of action for people with multiple sclerosis (pwMS). However, physicians need to be mindful of potential complications especially in patients undergoing anti CD20 and manage them appropriately.

## 1 INTRODUCTION

In December 2019, Wuhan, China, witnessed numerous cases of unexplained acute pneumonia. The swift and widespread transmission and severity of the situation quickly garnered international focus. Consequently, the genome sequence of the novel virus was recognized as severe acute respiratory syndrome Coronavirus 2 (SARS‐CoV‐2), giving rise to the COVID‐19 pandemic. As of July 2024, over 776 million confirmed cases have been reported as a result of this pandemic globally, which has led to over 7 million deaths.^1^ Various strategies were implemented to mitigate the severe impact of the virus, prompting pharmaceutical companies to expedite vaccine development to curb the rising infection rates. These eagerly anticipated vaccines were expected to decrease the frequency, severity, and disease‐related burden.^2^

**Figure 1:**
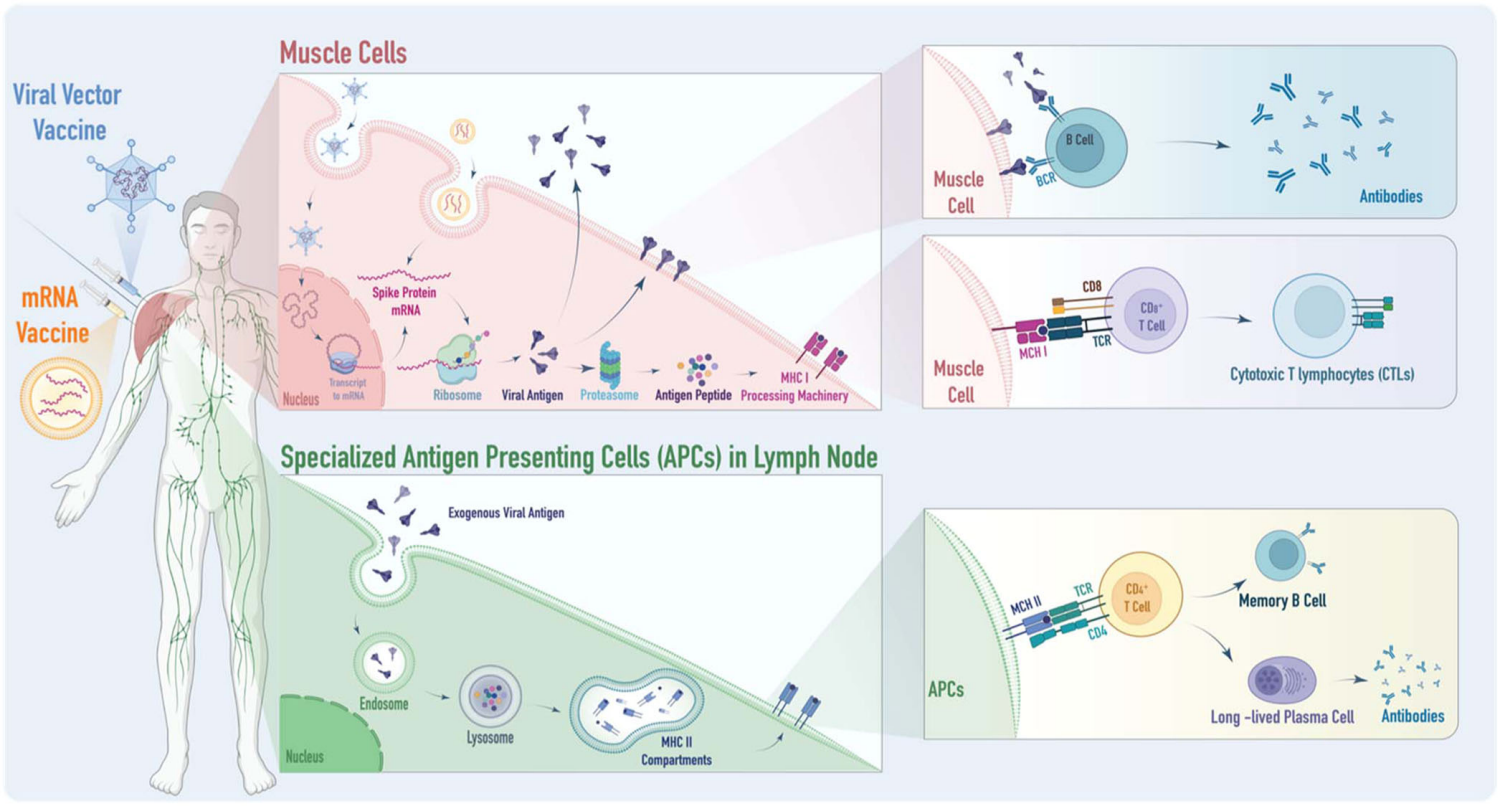
Depicts the immune response mechanisms triggered by two types of COVID‐19 vaccines: adenovirus vector and mRNA vaccines. The adenovirus vector vaccine introduces DNA into host cells, which is then transcribed into mRNA and translated into viral proteins. The mRNA vaccine directly delivers mRNA into cells, where it's translated into proteins. These proteins act as antigens, stimulating the immune system. Processed by proteasomes and antigen‐presenting cells, these antigens activate CD8 + T cells through MHC class I molecules and CD4 + T cells through MHC class II molecules. This activation leads to the destruction of infected cells and stimulates both cellular and humoral immune responses, including the activation of B cells and the release of cytokines, providing comprehensive immunity against the virus.

The urgent need for effective vaccines was highlighted by our increasing understanding of the virus's entry mechanism into human cells, primarily through the Angiotensin‐converting enzyme‐2 (ACE2) receptor, a critical target for vaccine‐induced antibodies. The receptor‐binding domain (RBD) of the SARS‐CoV‐2 spike (S) protein engages with the ACE2 receptor (Figure 1).^3^ Besides, for the virus to gain entry into the host cell, it's crucial that the viral S protein undergoes priming, which consists of the cleavage of the S protein. The process of cleavage is enabled by serine proteases, specifically transmembrane serine protease 2 (TMPRSS2).^4^

Given the rapid spread of the virus and its impact on numerous individuals, vaccination is the most effective method to halt the disease's transmission and reduce the ongoing pandemic.

Due to its extensive genetic diversity, SARS‐CoV‐2 has undergone several mutations, leading to the emergence of various virus variants. As a result, the process of developing vaccines becomes more complex, as mutations in the S protein can potentially impact the efficacy of vaccines, making it more challenging to ensure sufficient efficacy against these variants.^5^ Nevertheless, various types of COVID‐19 vaccines are available. All these vaccines are engineered to activate immunity to effectively identify and neutralize the SARS‐CoV‐2 virus.

The rapid development of COVID‐19 vaccines and the emergence of numerous viral mutations pose particular challenges for patients with chronic diseases such as Multiple Sclerosis (MS) patients. MS is a chronic inflammatory, demyelinating, and neurodegenerative disorder of the central nervous system (CNS) impacting both the white and gray matter of the brain, spinal cord, and optic nerve.^6^ It stands as the most prevalent cause of non‐traumatic neurological disability in individuals who are young and middle‐aged. The first signs usually manifest between the ages of 20 and 50, and women face a roughly threefold higher probability of developing MS in comparison to men.^7^ During the COVID‐19 pandemic, a significant concern has arisen about whether COVID‐19 has a distinct impact on individuals with MS. Additionally, questions have surfaced regarding any unique responses to vaccines among people with MS (pwMS), including the potential for MS relapse following vaccination.

Our goal is to scrutinize various types of COVID‐19 vaccines, assessing their efficacy, adverse events, the potential for MS relapse following vaccination, and the influence of disease modifying therapies (DMTs) on the vaccines’ efficacy.

## 2 METHODS

The database search included PubMed/Medline, Embase, Scopus, and the Web of Science and was conducted from January 2020 to July 2024 using the following MeSH terms: MS, COVID‐19, COVID‐19 Vaccination, and vaccine hesitancy. The following inclusion criteria were applied: 1. English‐language articles; 2. Peer‐reviewed studies; 3. Studies investigating the effectiveness of COVID‐19 vaccines in pwMS, the adverse effects of COVID‐19 infection in pwMS, the adverse effects of COVID‐19 vaccination in pwMS, and comparisons between the adverse effects of COVID‐19 infection and vaccination in pwMS. Two authors screened all titles and abstracts based on predetermined criteria. Full texts of studies meeting the inclusion criteria were subsequently screened using EndNote (X20).

## 3 COVID‐19 VACCINES

There have been multiple types of vaccines created and tested for SARS‐CoV‐2. These include traditional methods such as live attenuated, inactivated, protein/adjuvant approaches, viral vectors, and nucleic acids. In this review, we will focus on the most commonly used vaccines, which are composed of mRNA nucleic acid and viral vectors.

### 3.1 mRNA vaccine

#### 3.1.1 BNT162b2 vaccine (Pfizer‐BioNTech)

BNT162b2, which is a product of collaboration between BioNTech and Pfizer, is alternatively referred to as Pfizer‐BioNTech. This vaccine employs modified mRNA with nucleoside adjustments to encode the S glycoprotein of SARS‐CoV‐2, all contained within lipid nanoparticles.^8^ BNT162b2 demonstrated a 95% efficacy in preventing COVID‐19 when administered as a two‐dose injection. This level of efficacy remained consistent across various subgroups, including individuals of varying ages, genders, races, ethnicities, levels of obesity, and individuals already affected by medical issues.^9^ Common side effects include pain at the injection site, fever, chills, nausea, fatigue, muscle pain, headache, and joint pain. Some patients also experienced fever following both the first and second vaccine doses, although it usually subsided within a day.^10^

#### 3.1.2 mRNA‐1273 vaccine (Moderna)

**Figure 2:**
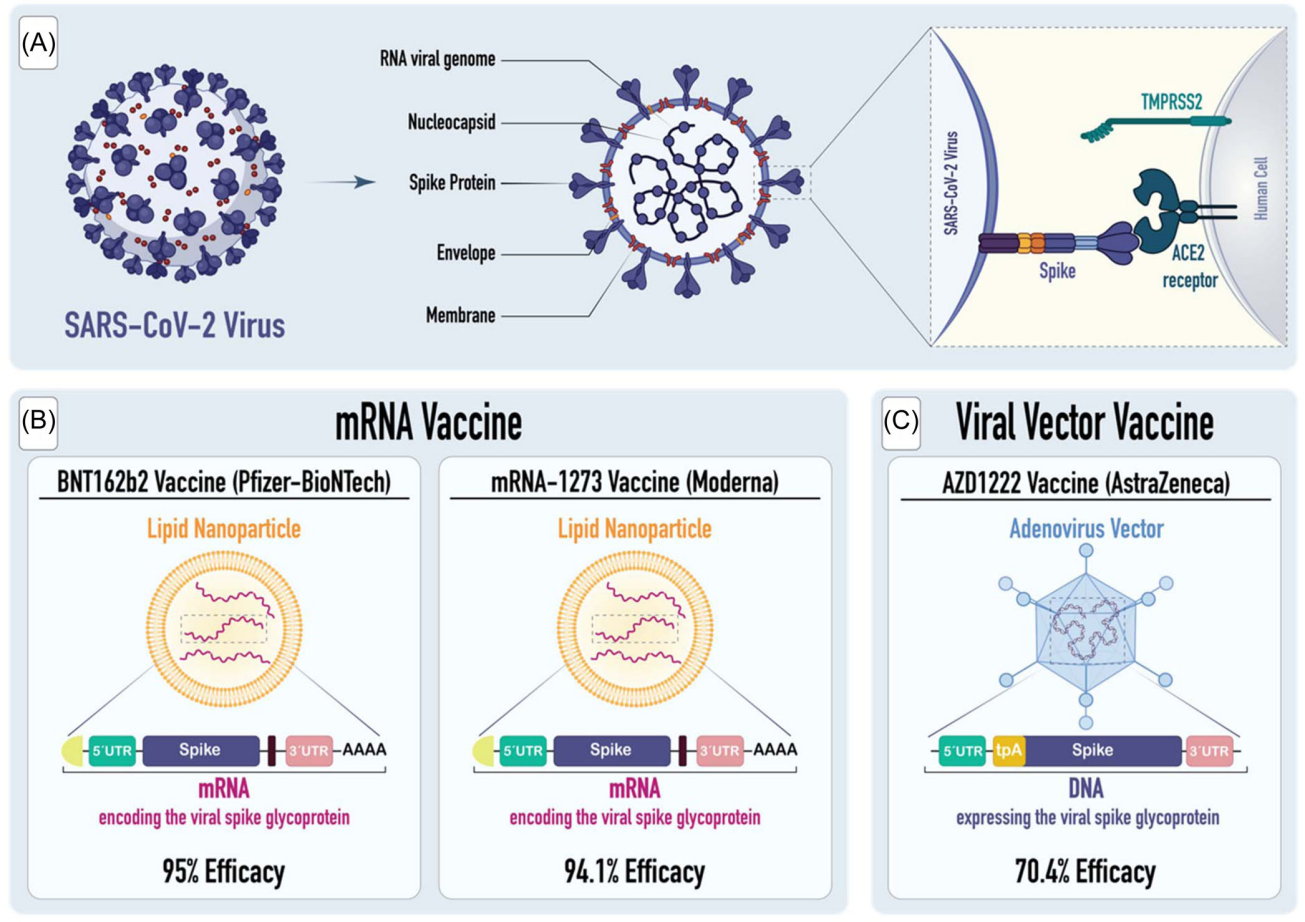
Schematic representation of SARS‐CoV‐2 structure and three different COVID‐19 vaccines. (A) SARS‐CoV‐2 is a virus characterized by its encapsulating lipid membrane and positive‐strand RNA. It possesses five primary structural components: the spike, membrane, envelope, its RNA genome, and the nucleocapsid proteins. The spike protein specifically engages with the angiotensin‐converting enzyme 2 (ACE2) via its receptor‐binding domain (RBD), and utilizes the transmembrane protease serine 2 (TMPRSS2) for cellular entry. (B) Regarding mRNA vaccines, they represent a novel vaccine technology that administers lipid nanoparticle carriers loaded with RNA sequences that encode for designated antigens. (C) Finally, adenovirus‐vectored vaccines are engineered through the fusion of a non‐replicating adenovirus vector with the desired DNA sequence, creating a recombinant vaccine.

mRNA‐1273 vaccine, created through a collaboration between the National Institutes of Health (NIH) and Moderna, utilizes modified mRNA to encode the spike glycoprotein of SARS‐CoV‐2 (Figure 2). Lipid nanoparticles are used as delivery vehicles for this vaccine.^11^ As measured by the efficacy rate of 94.1%, the vaccine prevented symptomatic infection with SARS‐CoV‐2.^12^ This level of efficacy remained consistent across all subgroups, including individuals aged 18 to < 65 and ≥ 65 years, those with comorbidities, as well as individuals of different genders, races, and ethnicities. Additionally, for participants with prior exposure to the SARS‐CoV‐2 and tested positive for antibodies, the vaccine showed an efficacy of 93.6%.^13^

The side effects are similar to those associated with the Pfizer vaccine, including injection site pain, as well as symptoms like fatigue, headache, muscle and joint aches, chills, and swollen lymph nodes in the arm where the vaccine was administered which typically resolve within a day or two.^14^ In addition to these common side effects, there have been reports of more serious adverse effects occurring in a small percentage of individuals who receive the vaccine.

The advantages of mRNA vaccines were quickly noticeable upon their initial administration to the general population, as their use was swiftly associated with a decrease in COVID‐19 symptoms and spread.^15^ The key advantage of mRNA vaccines is their ability to be rapidly modified by simply changing the immunogenic transgene. Therefore, the redesign of mRNA vaccines in response to new variants of SARS‐CoV‐2 occurs rapidly.^16^ Notable but rare serious adverse events associated with mRNA vaccines include reduced efficacy against specific variants and the activation of Th17 immune responses, which can exacerbate inflammatory reactions.^16^

### 3.2 Viral vector vaccine

#### 3.2.1 AZD 1222 vaccine (ChAdOx1 nCoV‐19, AstraZeneca)

The University of Oxford developed AZD 1222 (AstraZeneca), which is a vaccine created using genetically engineered chimpanzee adenovirus.^17^ This vaccine demonstrated a 70.4% efficacy against COVID‐19 in adults, 14 days after the second dose. Just like mRNA vaccines, common side effects associated with this vaccine include injection‐site reactions and pain, as well as headache, malaise, and nausea. These side effects typically resolve within a few days.^18^ However, there are occasional documented instances of significant negative reactions, including the emergence of vaccine‐induced immune thrombotic thrombocytopenia (VITT) and cerebral venous sinus thrombosis (CVT), subsequent to the administration of the vaccine.^19^

### 3.3 Humoral and cellular immune response following COVID‐19 vaccination

In immunological studies, T lymphocytes and B lymphocytes are shown to interact cooperatively within the adaptive immune system, which assists in the memory of the immune system and the production of neutralizing antibodies. An innate immune response occurs upon recognition of vaccine antigens.^20^ There is a significant immune response induced by vaccines against the SARS‐CoV‐2 spike protein on both the humoral and cellular levels. To estimate the protective antibody response to SARS‐CoV‐2, a quantitative measurement of antibodies targeting receptor‐binding domains is employed, as antibodies targeting these domains have been demonstrated to neutralize the infection.^21^ The immune response induced by vaccines is mediated by both B‐cells and T‐cells.^22^ There is no doubt that T cells play an important role in the production of antibody‐producing plasma cells, long‐lived memory cells, as well as in eradicating viruses. COVID‐19 infections have been associated with early and robust T‐cell responses, even in the absence of anti‐COVID‐19 antibodies.^23^ The precise combination of immune responses that provides the most robust immunity against SARS‐CoV‐2 is still uncertain. This holds true for both individuals without underlying health conditions and for those undergoing B and T cell‐depleting therapies.^24^

## 4 EFFECTS OF DMTS ON COVID‐19 VACCINATION

DMTs are a category of treatments designed to modify the course of MS. All the currently available DMTs for treating MS operate primarily by suppressing the activity of the immune system, which, in turn, mitigates the intensity of the inflammatory response responsible for the condition, and consequently reducing the risk of relapses by decreasing disease activity as observed on MRI scans, and/or slowing the progression of MS.^25^ The selection of DMTs for MS treatment is continually expanding. In the United States, there are over 25 DMTs that have received FDA approval for managing relapsing forms of MS.

DMTs have the potential to influence immune responses to SARS‐CoV‐2 vaccines.^26^, ^27^ Patients with a weakened immune system and those using specific medications or treatments are at a higher risk of experiencing severe complications from COVID‐19.^28^ DMTs also increase the risk of hospitalization and mortality following COVID‐19 infection in pwMS.^29^

**Figure 3:**
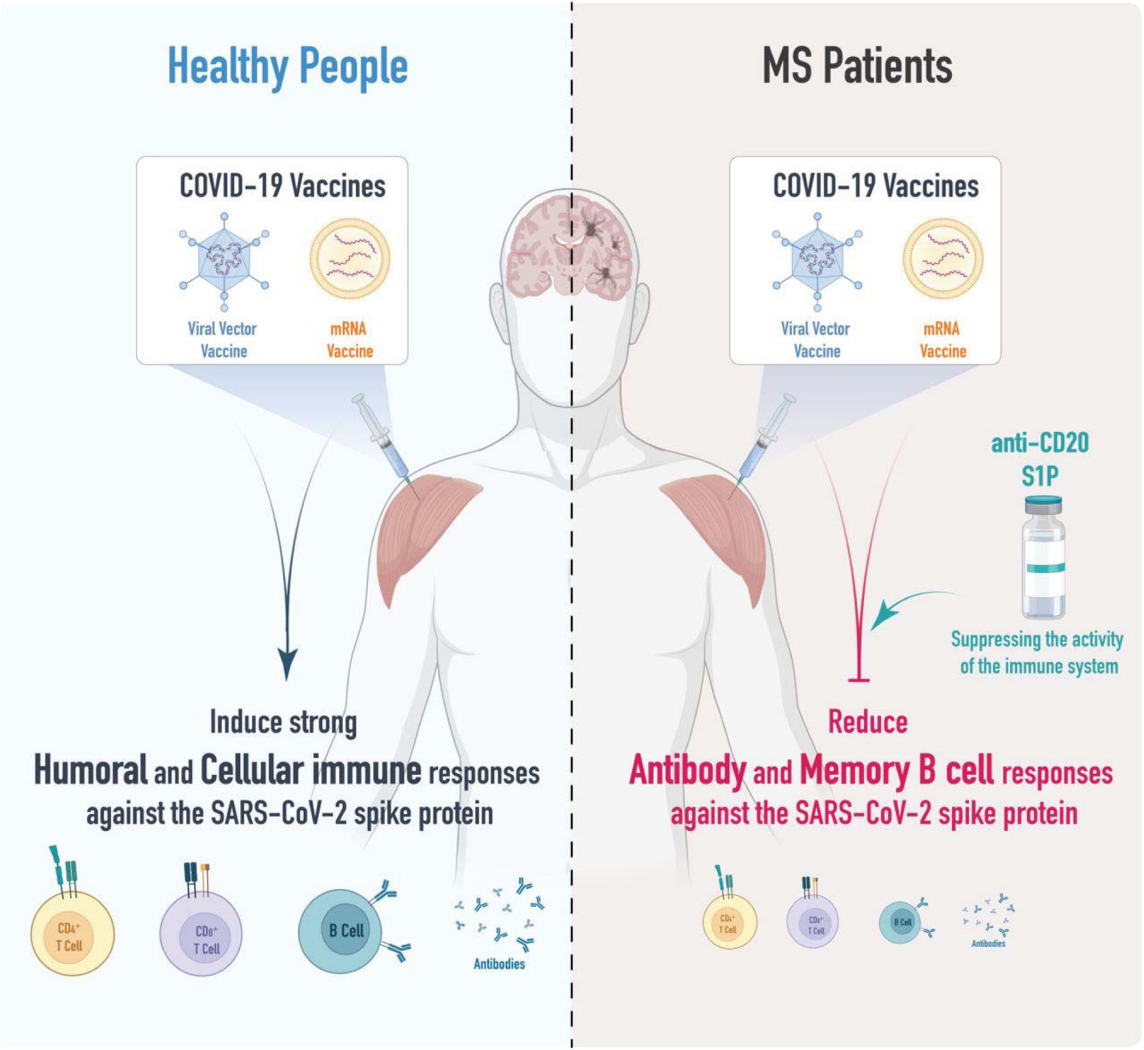
Diverse immune reactions to COVID‐19 vaccines in individuals with and without MS. MS patients, who are often prescribed disease‐modifying therapy (DMT) drugs, experience a suppression of their immune system, leading to reduced antigen response and blood immunity following COVID‐19 vaccination.

Among all DMTs, a notable reduction in the humoral response has been observed following COVID‐19 vaccination in patients who received anti‐CD20^30^ and Sphingosine‐1‐phosphate (S1P) receptor modulator drugs (Figure 3). Previous studies have reported a cellular response to COVID‐19 in seronegative patients who recently received anti‐CD20 therapy, suggesting some benefits despite the impaired B‐cell response.^31^, ^32^ Therefore, the T‐cell response to COVID‐19 vaccines presumably remains intact and can help reduce the complications of severe infection.^33^ Interestingly, a T‐cell response was detected after the third vaccine dose in some patients who did not achieve a humoral response, highlighting the importance of timely and up‐to‐date vaccination for vulnerable populations.^34^

Sormani et al. investigated the outcomes of pwMS who had suspected or confirmed COVID‐19. Their follow‐up study showed that patients treated with anti‐CD20 drugs had higher incidences of pneumonia, hospitalization, and severe complications compared to those on other DMTs.^35^ These findings were corroborated by a recent study by Feuth et al., which demonstrated that pwMS receiving anti‐CD20 drugs are more susceptible to prolonged viral pneumonia and increased mortality.^36^

Conversely, other types of DMTs were not significantly associated with impaired antibody responses in pwMS.^37^ Patients receiving anti‐CD20 therapies or S1P receptor modulators are more likely to develop COVID‐19, regardless of whether they have been vaccinated, as opposed to patients receiving other DMTs.^38^ Based on Jakimovski et al. study, it is typical for only 20% to 30% of individuals who receive these drugs to exhibit a detectable antibody response, which puts them at an elevated risk of experiencing breakthrough COVID‐19 infections.^39^

The duration of treatment and the development of antibodies have been found to be significantly related by Ciccone et al. Their findings indicate that a shorter treatment duration is positively associated with a higher probability of seroconversion, whereas a more extended treatment duration is concomitant with an elevated risk of experiencing a diminished antibody titer.^40^

These results were confirmed by Nytrova et al., who found that the duration of therapy in seroconverted patients was significantly shorter (averaging 2 years) compared to the nonresponse group.^41^ Tallantyre et al. conducted a comprehensive multicenter cohort study to investigate the influence of DMTs and vaccine types on the serological response to COVID‐19 vaccination. Among patients not receiving any DMT or those on natalizumab, alemtuzumab, dimethyl fumarate, cladribine, glatiramer acetate, interferon beta, and teriflunomide, those who received the BNT162b2 mRNA (Pfizer‐BioNTech) vaccine exhibited a significantly stronger IgG response to vaccination compared to those who received the ChAdOx1 nCoV‐19 (Oxford‐AstraZeneca) vaccine. However, there was no discernible difference in serological response based on vaccine type for individuals who had undergone treatment with anti‐CD20 monoclonal antibodies. It is noteworthy that anti‐CD20 monoclonal antibodies were the DMTs most closely associated with a lack of seroconversion following COVID‐19 vaccination. Additionally, the study revealed that fingolimod was linked to both a substantially reduced humoral response to COVID‐19 vaccination and a low count of T cell responders among those who did not respond to the vaccine.^42^

In a systematic review conducted by Gombolay and colleagues, it was revealed that mRNA vaccines displayed a higher likelihood of generating a response to the antibody in both healthy individuals and pwMS when compared to other vaccines, particularly inactivated viral vaccines. Among the various DMTs, some treatments demonstrated minimal impact on responses of the vaccine, notably beta‐interferons, glatiramer acetate, teriflunomide, dimethyl/diroximel fumarate, and natalizumab. Conversely, the most significant effects were observed in connection with anti‐CD20 monoclonal antibodies and S1P modulators, resulting in a decreased detection of SARS‐CoV‐2 antibodies. The majority of patients demonstrated evidence of vaccine‐induced antigen‐specific CD4+ and CD8 + T‐cell responses after receiving mRNA vaccines, even though B‐cell‐depleting therapies may reduce anti‐SARS‐CoV‐2 antibodies and memory B cell responses. T cells were primed as a result of the mRNA vaccine.^43^, ^44^

For pwMS who are presently undergoing DMTs, it is strongly recommended not to delay their SARS‐CoV‐2 vaccination if they are using interferons, teriflunomide, ofatumumab, natalizumab, dimethyl fumarate, glatiramer acetate and S1P drugs.^45^, ^46^ Concerning Cladribine, it is advisable to wait at least 4 weeks after the last course of therapy or, in cases of lymphopenia, until lymphocyte counts have recovered.^46^ Patients can start taking Cladribine tablets 2–4 weeks after getting vaccinated.^47^ However, for patients receiving ocrelizumab and rituximab, it is advised to postpone vaccination by at least 12 weeks after their last dose, and for those treated with alemtuzumab, a 6‐month delay after the last dose is recommended.^48^ Commencement of treatment with beta interferons, glatiramer acetate, teriflunomide, dimethyl fumarate, or natalizumab does not require postponement following COVID‐19 vaccination.^49^ For the initiation of treatment with S1P modulators, ocrelizumab, ofatumumab, and rituximab, it is advisable to delay it by 4 weeks after vaccination to maximize the response of the vaccine.^50^ When considering alemtuzumab, it should be initiated at least 4 weeks after vaccination.^45^ These recommendations are intended to ensure the optimal and safe integration of COVID‐19 vaccinations for individuals undergoing various MS treatments.^51^

## 5 PERCEIVED SAFETY, SIDE EFFECTS AND COMPLICATIONS OF COVID‐19 VACCINES IN PWMS

Like with any other vaccines, there has been some skepticism about the use of the COVID‐19 vaccines. In a comprehensive cross‐sectional study conducted in Australia among individuals affected by cancer, diabetes, and MS, significant rates of COVID‐19 vaccination adoption and a strong willingness to get vaccinated were observed in these patients. It should be noted that these trends seem to have been driven by concerns regarding how getting infected with SARS‐CoV‐2 might affect their underlying medical conditions. As a result of their diseases and associated treatments, these individuals are particularly vulnerable to infection, and vaccination may protect them from infection as well as improve their ability to manage their disease.^52^

When it comes to pwMS specifically, studies indicate that individuals who have access to sufficient information are more inclined to receive the COVID‐19 vaccinations.^53^ Moreover, patients tend to place greater trust in information when it is provided by healthcare professionals or National MS Society.^54^ In a comprehensive study investigating the intention of pwMS to receive the COVID‐19 vaccine, it was discovered that the overall willingness rate was 76%, with individual rates ranging from 49% to 94%. This demonstrates a high level of willingness among these patients to be immunized.^55^

Neurological adverse events that occur with any type of COVID‐19 vaccine can range from mild to severe, with some being life‐threatening.^56^ Severe CNS complications linked to COVID‐19 vaccines include a range of conditions, such as venous sinus thrombosis, intracerebral ischemic and hemorrhagic strokes, subarachnoid bleeding, cerebral vasoconstriction, and vasculitis.^57^ Furthermore, vaccine‐related complications that affect the CNS include inflammatory disorders such as encephalitis, meningitis, demyelinating disorders, and transverse myelitis. Additionally, there have been reported associations between these vaccines and epilepsy.^57^ Peripheral nervous system disorders related to COVID‐19 vaccines consist of various conditions such as cranial nerve neuropathy, Guillain‐Barre syndrome (GBS), small fiber neuropathy, myasthenia gravis, and facial palsy.^58^, ^59^ The occurrence of neurologic side effects following acute infection with COVID‐19 is as much as 617‐fold higher in comparison to post‐COVID vaccination scenarios.^60^ In addition, many cases of DVT were reported after the Janssen COVID‑19 vaccine.^61^ Furthermore, myocarditis and pericarditis are rare, yet significant side effects related to COVID‐19 vaccines, with incidence rates of 1.71 and 2.71 per 100,000 individuals for the mRNA‐1273 and BNT162b2 vaccines, respectively.^62^

A relapse is defined as the onset of new symptoms or symptoms similar to those from a prior relapse, which persist continuously for at least 24 h without any evidence of infection or significant change body temperature. Additionally, at least 30 days must have passed since the last relapse or symptom flare‐up.^63^ However, the duration can vary significantly, ranging from just a few days to several months. The severity of relapses can vary as well, with acute relapses at their most severe possibly necessitating hospital treatment.^64^ MS relapses are believed to be affected by many factors, such as age, gender, pregnancy, vitamin D levels, genetic influences, environmental factors, and infectious diseases.^65^

There is a belief that relapses linked to an infection may inflict greater neurological harm compared to relapses not connected to infections.^66^ Consequently, taking steps to avoid infections is especially crucial for individuals living with MS. SARS‐CoV‐2 infections have been associated with potential new‐onset MS^67^ and may also trigger relapses and pseudo‐relapses in pwMS.^68^ According to histopathological evidence, it can be deduced that COVID‐19 infection disrupts the structural integrity of the blood‐brain barrier, infiltrates specific regions within the CNS where MS lesions are located, and induces pathophysiological issues in pwMS who are already predisposed to such complications.^69^ Furthermore, there is a connection between COVID‐19 infection and the pathological cascade that can potentially trigger new‐onset or relapsing forms of neuromyelitis optica spectrum disorder. While the exact underlying mechanism is not fully understood, demyelinating alterations are thought to occur either as a result of a hyperinflammatory state triggered by infection, leading to the release of cytokines and subsequent glial activation, or as part of a delayed immune response.^70^

When it comes to vaccination, pwMS tolerate most of the vaccines and they do not have an adverse effect on the progression of the disease.^71^ The only exception involves live or live‐attenuated vaccines. The National MS Society advises against administering these vaccines to individuals with MS who have recently taken steroids or are on specific DMTs. Furthermore, Individuals undergoing a relapse should postpone vaccination until the symptoms of the relapse have ceased or are no longer worsening.^72^ A study conducted by Maniscalco et al. on flu vaccination in pwMS demonstrated that the flu vaccine is well tolerated among them, with the majority not experiencing serious adverse effects.^73^ However, an MS relapse may still occur following COVID‐19 vaccination, with sensory deficits being the most commonly reported symptoms during such relapses.^74^ It's important to understand whether the risk of an MS relapse is higher in vaccinated or unvaccinated patients. Therefore, Achiron et al. conducted a study on a group of pwMS who received the COVID‐19 vaccine and monitored them for the incidence rate of MS relapse. In this study, the first dose of the COVID‐19 vaccine was administered to 555 pwMS, and the second dose was given to 435 pwMS. During the follow‐up period, the rate of patients experiencing acute relapses was 2.1% after the first dose and 1.6% after the second dose. These rates were comparable to those observed in non‐vaccinated patients during the same timeframe.^75^

Numerous studies have examined the safety of the COVID‐19 vaccine in pwMS. In a recent cohort study, 208 pwMS were followed up regarding their side effects of COVID‐19 vaccines Pfizer‐BioNTech and Oxford‐AstraZeneca. About 70% of them experienced side effects, with most reactions being early onset, such as injection site pain or minor systemic side effects like headache, fever, and fatigue, which resolved spontaneously within a few days.^76^ This result aligns with findings from a study by Czarnowska et al., which included 2261 pwMS receiving the COVID‐19 vaccine, with an equivalent percentage experiencing mild, similar symptoms.^77^

In a study involving 13 pwMS in Korea to assess vaccine safety, only one patient experienced a relapse post‐vaccination, and no serious adverse events were reported. These findings, consistent with earlier research, indicate a low risk of relapse and serious adverse events following COVID‐19 vaccination in pwMS.^78^ These studies indicates the relative safety and efficacy of COVID‐19 vaccines in pwMS, with comparable rates of adverse reactions compared to the general population, irrespective of the use of commonly prescribed DMTs for pwMS, emphasizing the benefits of vaccination in this group.^76^

There is a limited number of investigations examining the safety and effectiveness of AstraZeneca vaccination in pwMS. A substantial study revealed that pwMS using DMTs, excluding anti‐CD20‐depleting therapies, who were administered ChAdOx1 nCoV‐19, exhibited notably lower IgG antibody levels in comparison to a group receiving the BNT162b2 mRNA vaccine under similar conditions.^42^ Additionally, there is documented evidence of new relapses confirmed via MRI scans occurred in 8 pwMS who were under DMTs treatment following their initial AstraZeneca vaccination dose.^79^ This emphasizes the importance of taking preventive steps when giving ChAdOx1 nCoV‐19 to pwMS.

## 6 CONCLUSION

In this review, we discussed the mechanisms of action of COVID‐19 vaccines in pwMS who are taking different types of DMTs and summarized adverse effects reported in these patients. In PwMS evidence supports that receiving the any type of COVID‐19 vaccine stands as a safer and more reliable approach to building immunity in contrast to being infected by the virus. The vaccines effectively trigger an immune response, affording protection without the inherent risks of severe illness or the potential postinfection complications associated with a COVID‐19 infection.

However, like any other vaccine, COVID‐19 vaccines can cause mild complications such as fatigue, headache, and injection site soreness, as well as severe complications like DVT, myocarditis, pericarditis, encephalitis, meningitis, and GBS. Besides, an MS relapse may still occur following COVID‐19 vaccination, but the risk of relapse is comparable to that in non‐vaccinated patients, indicating a low risk of relapse following vaccination. This suggests that COVID‐19 vaccination does not significantly increase the risk of MS relapse, providing reassurance for patients regarding the safety of vaccination. Physicians should be aware of the limited humoral response to vaccination in pwMS on anti‐CD20 therapies and S1P modulators, as well as the limited cellular response in those treated with S1P modulators. Despite these limitations, the benefits of vaccination still outweigh the risks for patients on anti‐CD20 therapies, given their higher risk of infection and potentially more severe disease course, according to available literature. It is also crucial to consider the appropriate timing of vaccination in relation to DMT administration, especially for therapies such as anti‐CD20, alemtuzumab, or cladribine. Therefore, the continuous and active promotion of immunization, particularly for pwMS, is essential and strongly recommended. It's crucial to counteract misinformation, as it can undermine adherence to guidelines.

## AUTHOR CONTRIBUTIONS

**Farhad Mahmoudi**: Conceptualization; Methodology; Investigation; Supervision; Writing—review and editing; Writing—original draft. **Omid Mirmosayyeb**: Writing—original draft; Writing—review and editing; Conceptualization; Investigation. **Elnaz Shaabani**: Visualization; Writing—review and editing; Software; Writing—original draft. **Elham Moases Ghaffary**: Writing—review and editing; Software; Validation. **Flavia Nelson**: Writing—review and editing; Project administration; Investigation; Conceptualization; Methodology; Supervision.

## CONFLICT OF INTEREST STATEMENT

The authors declare no conflicts of interest.

## TRANSPARENCY STATEMENT

The lead author Farhad Mahmoudi affirms that this manuscript is an honest, accurate, and transparent account of the study being reported; that no important aspects of the study have been omitted; and that any discrepancies from the study as planned (and, if relevant, registered) have been explained.

## Data Availability

The authors confirm that the data supporting the findings of this study are available within the article.
